# Shape and Morphology of the Sella Turcica in Patients with Trisomy 21—A Systematic Review

**DOI:** 10.3390/diagnostics16010022

**Published:** 2025-12-21

**Authors:** Magda Mazuś, Agnieszka Szemraj-Folmer, Marcin Stasiak, Michał Studniarek

**Affiliations:** 1 Department of Radiology, Faculty of Medicine, Medical University of Gdansk, 17 Smoluchowskiego Street, 80-210 Gdansk, Poland; michal.studniarek@gumed.edu.pl; 2 Department of Conservative Dentistry, Faculty of Medicine, Medical University of Gdansk, M. Sklodowska-Curie 3c, 80-210 Gdansk, Poland; agnieszka.szemraj-folmer@gumed.edu.pl (A.S.-F.); marcin.stasiak@gumed.edu.pl (M.S.)

**Keywords:** sella turcica, trisomy 21, Down syndrome

## Abstract

**Background/Objectives:** The sella turcica (ST) is a central craniofacial and endocrinological landmark whose morphology reflects both local skeletal development and systemic influences. Alterations in its form have been observed in various genetic syndromes, including trisomy 21 (Down syndrome, DS). Considering the characteristic craniofacial morphology of DS, this review aimed to evaluate whether individuals with DS present distinctive morphometric features and shape variants of the ST compared with non-syndromic populations and to discuss their diagnostic and clinical relevance. **Methods:** A systematic literature search was carried out in PubMed, the Cochrane Library, Web of Science, Wiley, MDPI, and Google Scholar on 8 May 2024. Search terms included “sella turcica,” “Down syndrome,” and “morphology.” Studies employing lateral cephalograms, cone-beam computed tomography (CBCT), or computed tomography (CT) to assess ST morphology were included when quantitative or qualitative comparisons with control groups were available. The review followed the PRISMA 2020 guidelines and was prospectively registered in PROSPERO (CRD42024580071). **Results:** Only six studies fulfilled the inclusion criteria. Increased ST dimensions and a predominance of U-shaped and J-shaped variants in individuals with DS compared with controls were most frequently reported. Although the studies differed in methodology, the findings consistently indicated characteristic enlargement and remodeling of the ST in trisomy 21. **Conclusions:** Individuals with Down syndrome exhibit distinctive sella turcica morphology characterized by increased size and specific shape variants. The evidence base remains small and heterogeneous, with few observational studies and mixed age groups and imaging modalities, which limits the strength and generalizability of the conclusions. The present study aims to provide a modern, updated systematic review of current evidence on sella turcica morphology in patients with Down syndrome, to identify reported patterns of variation, and to explore their clinical and diagnostic significance. Recognition of these features enhances diagnostic accuracy in craniofacial evaluation, facilitates comprehensive orthodontic, endocrine, and oncological assessment, and advances understanding of cranial base development within the context of genetic syndromes.

## 1. Introduction

The sella turcica (ST), a saddle-shaped depression in the sphenoid bone at the base of the skull, houses the pituitary gland and serves as a key anatomical landmark in craniofacial diagnostics [1,2]. In orthodontics, its clear visibility and stable position on lateral cephalometric radiographs make it a widely used reference point for evaluating craniofacial growth patterns and skeletal relationships [1]. Assessment of the sella turcica’s morphology may serve as the first indicator of underlying craniofacial, endocrine, or oncological abnormalities [1]. Variations in the size or contour of the ST have also been associated with several craniofacial and systemic disorders, as well as with pituitary tumors such as adenomas and other intracranial lesions [3,4].

**Figure 1:**
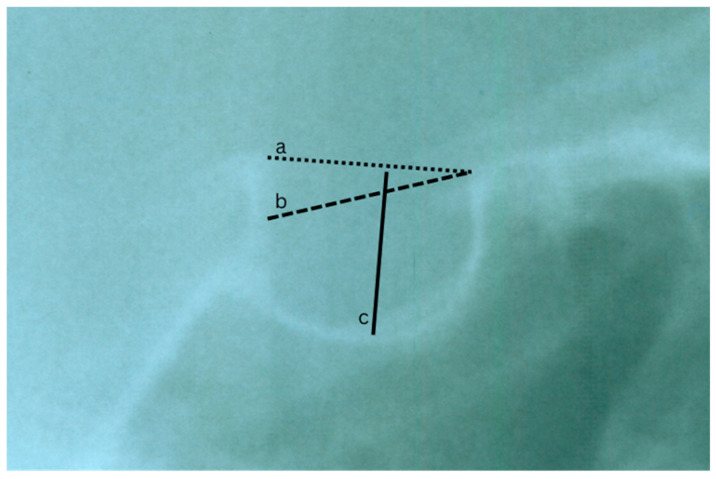
A lateral cephalogram showing the ST highlights its dimensions using the Silverman and Kisling method: (a) length: measured from the tuberculum sellae to the dorsum sellae; (b) depth: taken from the deepest point of the sella floor to a line connecting the tuberculum sellae and dorsum sellae; (c) diameter: represents the greatest distance between the superior and inferior borders.

Because lateral cephalograms form part of routine orthodontic records, clinicians may occasionally detect abnormalities in this region and, when necessary, facilitate timely referral for further medical assessment [5]. Several imaging modalities, including lateral cephalometric radiographs, cone beam computed tomography, and magnetic resonance imaging, are used to assess the size and morphology of the sella turcica [3,6,7,8]. Classifications proposed by Silverman and Kisling (based on size) and by Axelsson and Ruiz (based on morphology) provide useful frameworks for characterizing abnormalities in different populations [1,6,7]. The measurement protocol described by Silverman and Kisling is illustrated in Figure 1. Recent systematic reviews have further emphasized the relevance of ST morphology in craniofacial diagnosis and its variability across growth patterns and syndromic conditions [4,8].

Down syndrome (DS) is a genetic disorder characterized by intellectual disability, distinctive craniofacial features, and multiple systemic conditions [7,9,10,11]. Craniofacial anomalies commonly observed in DS include maxillary hypoplasia, reduced midface projection, and increased lower anterior facial height, which may also influence sella turcica morphology [9,10,11]. Investigating ST size and shape in individuals with DS may therefore provide valuable insights into the relationship between genetic syndromes, craniofacial development, and atypical sella morphology. Furthermore, abnormalities in this region may contribute to malocclusion in this population [9,10].

Despite several individual studies investigating sella turcica morphology in individuals with Down syndrome, the available evidence remains scarce, methodologically inconsistent, and fragmented across different imaging techniques and classification systems. Previous reviews of sella turcica morphology in broader populations have highlighted substantial variability in measurement protocols and shape definitions, as well as the absence of standardized diagnostic criteria [3,12]. However, no systematic review to date has focused specifically on individuals with trisomy 21, despite their unique craniofacial characteristics and the potential value of ST morphology as a supportive diagnostic feature. These factors indicate a clear need for a comprehensive and updated synthesis of the existing literature.

## 2. Materials and Methods

The study followed the Preferred Reporting Items for Systematic Reviews and Meta-Analyses (PRISMA) guidelines [13,14]. The PRISMA 2020 Checklist is provided in the Supplementary Materials.

The study protocol was registered with the Prospective Register of Systematic Reviews (PROSPERO) under the registration number CRD42024580071.

A narrative synthesis was conducted to integrate the findings from the various studies.

No deviations from the registered PROSPERO protocol were identified during the conduct of this review. All methodological steps, including study selection, data extraction, and risk-of-bias assessment, were carried out as originally planned.

### 2.1. Search Criteria

#### 2.1.1. Full Electronic Search Strategy

A comprehensive electronic search was conducted on 8 May 2024 in the following databases: PubMed, the Cochrane Library, Web of Science, Wiley Online Library, MDPI Journals, and Google Scholar. Only studies published in English were considered. The search strategy combined terms related to the sella turcica, Down syndrome, and morphological assessment. The exact search strings were adapted to the requirements of each database as follows: PubMed: (“sella turcica”[Title/Abstract] OR “sellar region”[Title/Abstract] OR “sella”[Title/Abstract] OR “sellar”[Title/Abstract] OR “sella turcica area”[Title/Abstract]) AND (“Down syndrome”[MeSH Terms] OR “Down syndrome”[Title/Abstract] OR “trisomy 21”[Title/Abstract]) AND (“morphology”[Title/Abstract] OR “morphometrics”[Title/Abstract] OR “shape”[Title/Abstract] OR “structure”[Title/Abstract] OR “dimensions”[Title/Abstract] OR “size”[Title/Abstract] OR “contour”[Title/Abstract] OR “malformations”[Title/Abstract]); Cochrane Library: (sella turcica OR sellar region OR sella OR sellar) AND (Down syndrome OR trisomy 21) AND (morphology OR morphometrics OR shape OR structure OR dimensions OR size OR malformations); Web of Science: TS = (“sella turcica” OR “sellar region” OR sella OR sellar) AND TS = (“Down syndrome” OR “trisomy 21”) AND TS = (“morphology” OR “morphometrics” OR “shape” OR “structure” OR “dimensions” OR “size” OR “malformations”); Wiley Online Library: (“sella turcica” OR “sellar region” OR sella OR sellar) AND (“Down syndrome” OR “trisomy 21”) AND (morphology OR morphometrics OR shape OR structure OR dimensions OR size OR malformations); MDPI Journals: “sella turcica” AND “Down syndrome” AND (morphology OR shape OR morphometrics OR dimensions); Google Scholar: “sella turcica” “Down syndrome” morphology OR morphometrics OR shape OR dimensions. Equivalent adjustments were applied where the database interface required. All retrieved records were exported to a reference management program for duplicate removal before screening.

#### 2.1.2. Inclusion Criteria

The PICO (Population, Intervention or Exposure, Comparison, Outcome) framework served as a template for constructing clinical questions. Characteristics of the studies:

P. Individuals with trisomy 21 (Down syndrome).
I. Radiological examination of sella turcica (lateral cephalograms, cone-beam computed tomography scans).
C. Comparison of sella turcica morphology between individuals with trisomy 21 and non-syndromic controls.
O. Evaluation of sella turcica morphology, including size, shape, and any deviations present in individuals with trisomy 21.

Only studies conducted in English and involving human subjects were considered.

#### 2.1.3. Exclusion Criteria

Articles not meeting the PICO criteria were excluded from the systematic review.

### 2.2. Data Collection

Data were extracted from the retrieved records for detailed text evaluation. These procedures were conducted by the first author, with the second author participating in cases of disagreement. The following information was collected: author, year of publication, country where the study was performed, study design, radiographic examination method, sample size, age range, male-to-female ratio, aim of the study, key findings, assessed parameters of the sella turcica, sella turcica morphometric results, conclusions, and limitations. Duplicate records, as well as letters and papers that did not contain significant information, were excluded.

### 2.3. Quality Assessment

In this article, we assessed the risk of bias in the included studies using the ROBINS-I tool (Risk of Bias in Non-randomized Studies of Interventions) and the Newcastle-Ottawa Scale (NOS). The ROBINS-I tool evaluates several domains: (1) confounding; (2) participant selection; (3) intervention classification; (4) deviations from intended interventions; (5) missing data; (6) outcome measurement; and (7) selection of reported results [14]. Each domain’s risk of bias was categorized as low, moderate, serious, critical, or no information available, with an overall risk score provided using the same gradation [15].

The Newcastle-Ottawa Scale assesses (1) study selection, (2) comparability, and (3) exposure. The quality of the studies is rated as follows: Good quality—3 or 4 stars in the selection category, 1 or 2 stars in the comparability category, and 2 or 3 stars in the exposure category; Fair quality—2 stars in the selection category, 1 or 2 stars in the comparability category, and 2 or 3 stars in the exposure category; Poor quality—0 or 1 star in the selection category, 0 stars in the comparability category, or 0 or 1 star in the exposure category [16].

## 3. Results

### 3.1. Literature Search

The electronic search identified 623 records, and no additional articles were retrieved through manual searching. After removal of duplicates, 612 unique records remained for screening. Of these, 604 records were excluded based on title and abstract review. Eight full-text articles were assessed for eligibility, and six studies met the inclusion criteria and were incorporated into the qualitative synthesis [16,17,18,19,20,21].

**Figure 2:**
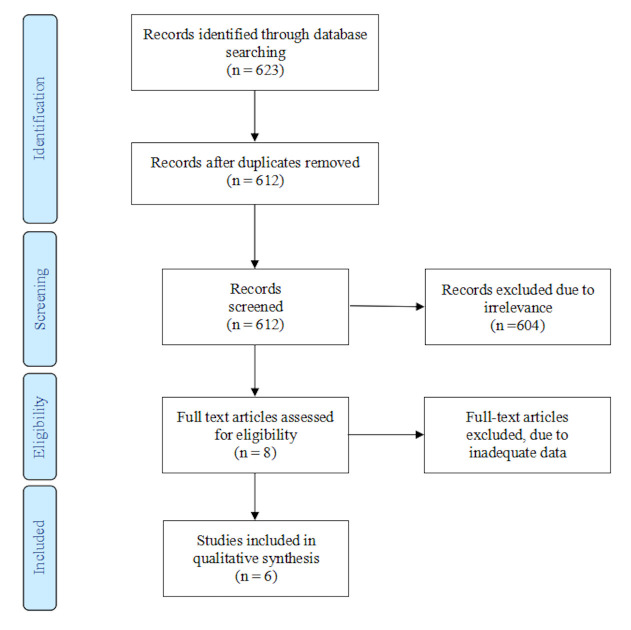
PRISMA flow diagram depicting the process followed for the selection of the studies.

The study selection process is illustrated in Figure 2.

### 3.2. Study Characteristics

The six included studies varied in design, geographical origin, imaging modality, and analytical approach. An overview of the studies is provided in Table 1. Four studies were observational in design [16,18,19,20], and two were retrospective analyses of previously collected radiographic or histological material [17,21]. The studies were conducted in Denmark [16,17], Saudi Arabia [18], Malaysia [19], Nigeria [21], and Turkey [20]. Sample sizes ranged from 22 fetuses in the prenatal study by Kjær et al. [17] to 78 postnatal participants in the study by Russell and Kjær [16]. Imaging methods included lateral cephalometric radiographs in four studies [16,18,20,21], histological and radiographic assessment in one fetal study [17], and computed tomography (CT) in one study [19]. The age range across studies encompassed fetal gestational ages of 14–21 weeks [17], children and adolescents, and adults up to 50 years [16].

Morphometric assessments included linear measurements such as height, depth, length, diameter, and area, depending on imaging modality. Shape assessment methods varied notably: some studies applied author-defined categories [16,17,21], one used a standardized orthodontic classification [18], one employed a structured set of CT-based shapes [19], and one used geometric morphometric analysis (Procrustes superimposition and Principal Component Analysis) [20].

### 3.3. Risk of Bias

In the present review, the risk of bias in the included studies was evaluated using two established tools: the ROBINS-I tool (Risk of Bias in Non-randomized Studies of Interventions) and the Newcastle-Ottawa Scale (NOS).

#### 3.3.1. ROBINS-I

Across the six included studies, two were evaluated as having a serious risk of bias, primarily due to the absence of appropriately matched control groups and limited methodological detail [16,17]. Two studies demonstrated a moderate risk of bias, mostly due to inconsistencies in measurement protocols and incomplete reporting of reliability [18,21], while the remaining two studies were judged to have a low risk of bias based on clear methodology, adequate control groups, and documented reliability procedures [19,20]. The principal sources of bias were the lack of adjustment for potential confounders, retrospective or cross-sectional study designs, and insufficient reporting of measurement reliability. A summary of ROBINS-I ratings is presented in Table 2.

#### 3.3.2. Newcastle-Ottawa Scale (NOS)

The NOS assessment further indicated methodological variability among the included studies. Two studies demonstrated serious limitations in selection and comparability domains, largely due to the lack of appropriate control groups and limited adjustment for potential confounders such as co-existing craniofacial anomalies [16,17]. Studies rated as moderate in quality typically reflected partial comparability or incomplete reporting [18,21], while two studies achieved a good-quality rating owing to well-defined samples, suitable control groups, and clearly described outcome measures [19,20]. NOS results are presented in Table 3.

## 4. Discussion

The studies included in this review report a wide range of findings regarding sella turcica morphology in individuals with Down syndrome. Several investigations identified differences in selected dimensions or shape characteristics when individuals with Down syndrome were compared with non-syndromic participants, whereas others, particularly those without control groups, described morphology that fell within the expected range for their samples. The variation in results reflects substantial methodological differences among studies, and no single pattern of sella turcica morphology can be considered characteristic across all six investigations.

### 4.1. Findings of Individual Studies

Kjær et al. examined twenty-two fetuses with trisomy 21 and described four characteristic morphological types of the sella turcica region [17]. Most fetuses displayed morphology considered close to typical, while a smaller subset showed anterior alterations, sometimes accompanied by axial skeletal anomalies. No dimensional comparisons were performed, and no control group was included.

Russell and Kjær evaluated seventy-eight postnatal individuals with Down syndrome and classified the sella turcica into three morphological types [16]. The majority showed an almost typical configuration, with deviations in the anterior wall or the floor observed less frequently. These findings were reported as broadly consistent with their earlier prenatal study.

Korayem and AlKofide assessed sixty individuals with Down syndrome and sixty matched controls using cephalometric measurements [18]. They reported statistically significant differences in sella depth and diameter between groups, while length did not differ significantly. Normal shape was less common in the Down syndrome group, which more frequently exhibited deviations such as an oblique anterior wall, sella bridging, and irregular posterior contour.

Hasan et al. compared fifty individuals with Down syndrome and fifty controls using computed tomography [19]. Several linear measurements differed significantly between groups, although not all parameters showed this pattern. U-shaped and J-shaped configurations were the most frequent shapes in the Down syndrome sample.

Aghimien evaluated twenty-nine individuals with Down syndrome and twenty-five additional subjects for shape assessment [21]. The study reported values for length, depth, and diameter, and the most common non-typical form was a pyramidal dorsum. When compared with published reference values, depth and diameter appeared greater, although the study did not include its own control group.

Papaefthymiou and Özbilén compared twenty-four individuals with Down syndrome and forty-eight matched controls [20]. They observed significantly greater values for sella height measures, length, and area in the Down syndrome group. Geometric morphometric analysis demonstrated a statistically significant difference in overall sella shape between groups.

### 4.2. Cross-Study Comparison and Conflicting Results

Among the three studies that included control groups, each identified statistically significant group differences in at least some sella turcica measurements [18,19,20]. However, the affected measurements varied between studies, and no consistent pattern was observed across all parameters.

In contrast, the two descriptive studies by Kjær and colleagues reported predominantly typical morphology in most individuals with Down syndrome [16,17]. The Aghimien study noted differences only in comparison with external normative data and did not include its own control group [21].

Shape findings were similarly inconsistent. Some studies reported increased frequencies of U- and J-shaped forms, others described pyramidal or author-defined types, and the prenatal and postnatal descriptive studies identified predominantly typical configurations [16,17,18,19,20,21]. Because shape classifications varied markedly, no unified morphological trend can be identified.

### 4.3. Sex-Related and Age-Related Findings

Sex-related findings varied. Some studies reported larger measurements in males [18], while others observed sex-related differences in specific measurements or shape patterns [21]. These results were based on small subgroups and remain inconclusive.

Age-related findings were also inconsistent. One study observed greater differences in younger individuals when Down syndrome and control groups were compared [19], while another study limited to adolescents found no such pattern [18]. The absence of longitudinal data prevents further interpretation.

### 4.4. Clinical Implications

Standardized evaluation of sella turcica morphology may support its use as a supplementary marker in fields such as pediatrics, endocrinology, and oncology in the future. However, as this remains a relatively novel area of investigation, its current clinical utility in individuals with Down syndrome is limited.

The studies included small samples, used different imaging methods, applied non-uniform measurement protocols, and relied on varying shape classifications.

These factors restrict comparability and limit the ability to draw clinically meaningful conclusions.

At present, sella turcica morphology should be regarded as a descriptive radiographic observation rather than a diagnostic or prognostic marker.

Future research should employ prospective standardized imaging methods and validated measurement and shape classification systems applied to larger, well-defined cohorts. Only with methodological consistency can the potential clinical significance of sella turcica morphology be evaluated reliably.

### 4.5. Limitations

Interpretation of this review is constrained by the small number of available studies, substantial heterogeneity in methodology, absence of control groups in two investigations, small sample sizes, broad age ranges, incomplete reporting of reliability, and the predominance of cross-sectional data. These limitations reduce confidence in any synthesis and restrict conclusions to the descriptive findings presented in the primary studies.

## 5. Conclusions

Several studies indicate a tendency toward increased sella turcica dimensions and a higher prevalence of characteristic U- and J-shaped variants in individuals with Down syndrome.

This review contributes to the field by systematically consolidating fragmented data and delineating recurring morphometric patterns reported across diverse study designs. However, no definitive morphological profile or confirmed clinical relevance can currently be established.

Larger, rigorously designed prospective studies employing standardized imaging and measurement protocols are needed to validate these preliminary trends and to clarify their potential diagnostic or clinical significance.

Although the existing evidence is limited and methodologically heterogeneous, the reported observations offer initial insights into craniofacial development in trisomy 21 and should be interpreted with appropriate caution.

## Figures and Tables

**Table 1:** The studies included in the qualitative analysis and extracted data.

**Reference**	Methodology	Study Population	ST Measurements	Shape Classification	Key Findings	Limitations
Kjær et al. 1998 Denmark [17]	Retrospective Histological and radiological	22 DS human fetuses (GA 14–21 weeks)	Qualitative assessment of the ST region	Author-defined (4 morphological types)	Minor to marked anterior sella changes; associated axial anomalies	No control group; very small sample; subjective classification
Russell and Kjær 1999 Denmark [16]	Observational Lateral cephalograms	78 DS; 4 months–50 years	Qualitative description	Author-defined (3 shape types)	Majority with near-normal morphology; few anterior/floor deviations	No control group; subjective classification
Korayem and AlKofide 2015 Saudi Arabia [18]	Observational Lateral cephalograms	60 DS, 60 controls 12–22 years	Linear: length, depth, diameter	Standardized (Axelsson classification)	DS individuals showed increased depth and diameter and more shape deviations	Small sample; no longitudinal data
Hasan et al. 2019 Malaysia [19]	Observational CT scan	50 DS, 50 controls; 0–35 years	Linear: heights (A/M/P), length, width, diameter, area	Modified standardized morphological criteria (U, J, shallow)	Several dimensions differed significantly; DS had U/J predominance	Sample limited to one population; generalizability limited
Aghimien 2022 Nigeria [21]	Cross-sectional descriptive Lateral cephalograms	29 DS (size), 25 DS. 10–20 years	Linear: length, depth, diameter	Author-defined	Shorter length; pyramidal shape common; sex-related variation	Small sample; no control group; indirect norm comparison
Papaefthymiou et al. 2023 Turkey [20]	Retrospective Lateral cephalograms	24 DS, 48 controls. 8–13 years	Linear: heights, length, width, area; geometric shape analysis	Standardized morphometric analysis (Procrustes + PCA)	DS group showed increased dimensions and distinct shape patterns	Small sample; no advanced imaging

**Table 2:** Risk of bias assessment using ROBINS-I tool.

Reference	Confounding	Selection of Participants	Classification of Interventions	Deviations from Intended Interventions	Missing Data	Measurement of Outcomes	Selection of Reported Result	Overall Risk of Bias
Kjær et al. 1998 [17]	Serious	Serious	Low	Low	Low	Moderate	Moderate	Serious
Russell and Kjær 1999 [16]	Serious	Serious	Low	Low	Low	Moderate	Moderate	Serious
Korayem and AlKofide 2015 [18]	Moderate	Low	Low	Low	Low	Moderate	Low	Moderate
Hasan et al. 2019 [19]	Low	Low	Low	Low	Low	Low	Low	Low
Aghimien 2022 [21]	Moderate	Moderate	Low	Low	Low	Moderate	Moderate	Moderate
Papaefthymiou et al. 2023 [20]	Low	Low	Low	Low	Low	Low	Low	Low

**Table 3:** Risk of bias using the Newcastle-Ottawa Scale for quality assessment.

Reference	Representativeness of the Exposed Cohort (S)	Selection of the Non-Exposed Cohort (S)	Ascertainment of Exposure (S)	Demonstration that Outcome Was Not Present at Start (S)	Comparability of Cohorts on Design or Analysis (C)	Assessment of Outcome (O)	Was Follow-Up Long Enough for Outcomes to Occur (O)	Adequacy of Follow-Up of Cohorts (O)	Total Score
Kjær et al. 1998 [17]	★	-	★	★	★	★	-	★	6/9
Russell and Kjær 1999 [16]	★	-	★	★	★	★	-	★	6/9
Korayem and AlKofide 2015 [18]	★	★	★	★	★★	★	★	★	8/9
Hasan et al. 2019 [19]	★	★	★	★	★★	★	★	★	9/9
Aghimien 2022 [21]	★	-	★	★	★	★	-	★	6/9
Papaefthymiou et al. 2023 [20]	★	★	★	★	★★	★	★	★	8/9

★ Low risk of bias; ★★ Moderate risk of bias, as assessed using the ROBINS-I (Risk Of Bias In Non-randomized Studies of Interventions) tool.

## Data Availability

The data presented in this study are available on request from the corresponding author. The data are not publicly available due to privacy restrictions.

## Supplementary Materials

The following supporting information can be downloaded at: https://www.mdpi.com/article/10.3390/diagnostics16010022/s1, PRISMA 2020 Checklist.

## Abbreviations

The following abbreviations are used in this manuscript:

ST	Sella Turcica
DS	Down Syndrome
CT	Computed Tomography
GA	Gestational Age
